# Combination of fecal SDC2 methylation with serum CEA/CA72–4 in screening of colorectal cancer and precancerous lesions

**DOI:** 10.1016/j.clinsp.2026.100987

**Published:** 2026-04-28

**Authors:** Zhen Li, Jianping Cheng, Xiaolin Zhao, Yang Liu, Yanqiong Zhang, Yanping Li, Yixiao Zhao, Chanjuan Fan

**Affiliations:** Department of Gastroenterology, Civil Aviation General Hospital, Beijing, China

**Keywords:** Colorectal cancer, Carcinoembryonic antigen, CA72–4, SDC2 Methylation

## Abstract

**Objective:**

To explore the application value of fecal Syndecan-2 gene Methylation (mSDC2) detection, combined detection of serum Carcinoembryonic Antigen (CEA) and Carbohydrate Antigen 72–4 (CA72–4) in screening of Colorectal Cancer (CRC) and precancerous lesions.

**Methods:**

A total of 196 participants were enrolled in this case-control study from March to December 2023, including 65 with CRC, 38 with advanced adenomas, 33 with non-advanced adenomas, and 60 controls. The sensitivity, specificity, and Odds Ratios (OR) for serum CEA, CA72–4, and fecal mSDC2 were evaluated.

**Results:**

The sensitivity of fecal mSDC2 for CRC was 86.2% (56/65), with a specificity of 96.7% (58/60), with an OR of 28.9 (95% CI 8.6–97.2, p < 0.001). The sensitivity in advanced adenomas was 34.2% (13/38). Serum CEA had a sensitivity of 56.9% (37/65) and a specificity of 96.7% (58/60) for CRC, with an OR of 5.7 (95% CI 2.0–16.4, p < 0.001). Combined detection of CEA and CA72–4 had a sensitivity of 69.2% (45/65) and a specificity of 81.6% (49/60). The triple combination (mSDC2 + CEA + CA72–4) achieved a sensitivity of 96.9% (63/65) and a specificity of 78.3% (47/60) in the CRC, and a sensitivity of 50% (19/38) for advanced adenomas. The combined detection had higher sensitivity than single detection, with statistically significant differences compared to serum-based detection (p < 0.001).

**Conclusion:**

Combining fecal mSDC2 with serum CEA and CA72–4 increased sensitivity for CRC detection in this single-center study, at the cost of reduced specificity. Validation in larger, screening-intended cohorts with predefined thresholds is warranted.

## Highlights

• A triple-marker strategy synergizes fecal DNA and serum tests for CRC screening.
• This combination achieves near-complete (96.9%) sensitivity for colorectal cancer.
• Half of precancerous advanced adenomas are identified by the combined approach.
• The method surpasses standard blood tests in early detection capability.

## Introduction

Colorectal Cancer (CRC) is one of the most significant malignant tumors that severely threatens human life and health worldwide.^1^ According to the latest statistical data, CRC ranks third in global incidence and second in mortality.^2^ Early diagnosis and treatments are critical for improving patient survival and quality of life.^3^ However, the 5-year survival rate of CRC in China remains lower than that in countries such as Japan and South Korea, as well as in Europe and the United States.^4^ A substantial body of clinical evidence shows that the 5-year relative survival rate of patients with early CRC can reach over 90% after timely and standardized treatment, while the 5-year survival rate of patients with advanced CRC drops to approximately 14%.^5^ Therefore, effective early screening can improve the survival rate of CRC patients and improve prognosis, which can significantly reduce the disease burden of CRC and enhance the long-term quality of life of patients.^6^

At present, the commonly used screening and diagnostic methods for CRC include the fecal occult blood test, serum tumor protein marker detection, and colonoscopy, among others.^7^ However, these traditional screening methods have revealed numerous shortcomings in practical applications. For instance, the fecal occult blood test has a relatively high false-positive rate, which can be easily influenced by diet and non-gastrointestinal bleeding factors.^8^ Serum tumor protein markers, such as Carcinoembryonic Antigen (CEA), have low sensitivity and cannot be used as reliable indicators for early diagnosis alone.^9^ Other serum markers, such as Carbohydrate Antigen 72–4 (CA72–4), have also been investigated for CRC. While CA72–4 alone demonstrates limited diagnostic power, it has been reported to exhibit complementary value when combined with CEA, particularly in identifying certain histological types or advanced stages of CRC, as it may reflect different tumor biological behaviors.^10^ This provides a rationale for exploring its combination with other modalities. Although colonoscopy is considered the gold standard for CRC diagnosis, its invasive nature may cause patient discomfort, leading to poor compliance and thus limiting the widespread implementation of screening.^11^

Given the limitations of existing screening methods, the exploration of highly sensitive, non-invasive, and patient-compliant detection methods has become an urgent need in the field of early CRC diagnosis. In recent years, fecal DNA testing technology has gradually attracted attention. This technology is based on the analysis of genetic and epigenetic characteristics of shed cancer cells in feces, and it offers high specificity and sensitivity, providing a new approach for the early screening of CRC.^12^ Among these, SDC2 methylation (mSDC2), as one of the key molecular events in the development of CRC has emerged as an innovative marker of interest in recent years. Multiple studies have confirmed that fecal mSDC2 testing can effectively detect CRC and advanced precancerous lesions.^13^, ^14^, ^15^

This study proposes the combination of fecal mSDC2 detection with the tumor markers CEA and CA72–4 detection, aiming to construct a more efficient diagnostic strategy for CRC. While the Fecal Immunochemical Test (FIT) serves as the current first-line non-invasive screening tool in many guidelines, its sensitivity, particularly for precancerous lesions, remains suboptimal.^16^ The exploration of biomarker combinations, such as integrating stool DNA methylation with serum protein markers, may offer a complementary approach by capturing different aspects of tumorigenesis, thereby providing a more reliable basis for clinical diagnosis.

## Methods

### Research design and sample sources

This case-control study adhered to the applicable 2007 Strengthening the Reporting of Observational Studies in Epidemiology (STROBE) guidelines. The study protocol was approved by the Institutional Ethics Board of the Civil Aviation General Hospital, Beijing, China (n°2023-L-K-07). Due to the retrospective design of the study and the fact that the study evaluated clinical practice, the Institutional Ethics Board of the Civil Aviation General Hospital waived the need for individual informed consent.

Data of patients were collected by using the electronic medical record system from March 2023 to December 2023 at the Department of Gastroenterology in the present study’s institution. A total of 196 participants were included: 65 patients with CRC, 38 with Advanced Adenomas (AA, characterized by any of the following features: high-grade dysplasia, adenomas with a diameter of ≥ 10 mm, villous adenomas, or tubulovillous adenomas), 33 with Non-Advanced Adenomas (N-AA, serrated lesions, adenomas or polyps with a diameter of < 10 mm), and 60 healthy controls. All of whom were confirmed by colonoscopy and pathology.

### Inclusion criteria

1) Age 18‒79 years; 2) No history of malignant tumors; 3) No radiotherapy, chemotherapy, or immunotherapy within the past 3-months; 4) Clear diagnosis confirmed by colonoscopy and pathology (no abnormalities found in colonoscopy for the healthy control group); 5) Willingness to sign the informed consent form and cooperate in providing stool samples.

### Exclusion criteria

1) Presence of mental disorders or cognitive impairment; 2) Severe dysfunction of the heart, lungs, liver, kidneys, or immunodeficiency; 3) Presence of other systemic malignant tumors; 4) Use of anticoagulant drugs or antibiotics within the past month; 5) Insufficient or improperly stored stool sample collection.

### Serum tumor marker detection

Collect 4 mL of fasting venous blood from the subject. The sample was centrifuged at 3000 r/min for 15 mins, then the serum was separated. Use electrochemiluminescence immunoassay (Roche Diagnostics CEA Elecsys and CA72–4 Cobas) to detect CEA and CA72–4 levels via an automated immunoanalyzer (Cobas e601, Roche Diagnostics). Positive threshold: CEA > 4.5 μg/mL or CA72–4 > 6.9 U/mL.

### Detection of SDC2 gene methylation in feces

The methylation detection procedure was strictly carried out according to the manufacturer's instructions, as described in the literature.^17^ Approximately 4.5 g of fecal sample was collected using the accompanying fecal collection device (registration number: YueSuXieBei 20,160,241). The sample was placed into the preservation solution and stored at room temperature, with the requirement of being sent for testing within 24-hours. DNA was extracted using the magnetic bead method (Human SDC2 Gene Methylation Detection Kit, National Medical Device Registration n°20,183,400,506). The unmethylated cytosines were converted to uracil through bisulfite treatment. The ABI 7500 instrument (Applied Biosystems) was used to amplify the target genes (SDC2 and the internal reference gene ACTB) by quantitative PCR. The reaction conditions were as follows: initial denaturation at 95 °C for 5-min; followed by 45 cycles of 95 °C for 15 s, 58 °C for 30 s, and 72 °C for 30 s. A test was considered positive if ACTB Ct ≤36 and SDC2 Ct ≤38, negative if ACTB Ct ≤ 36 and SDC2 Ct > 38, and invalid if ACTB Ct >36.

For the combined detection, a parallel testing strategy was employed. A participant was classified as screen-positive if the fecal mSDC2 test was positive OR the serum CEA level was above the cutoff (> 4.5 µg/mL), OR the serum CA72–4 level was above the cutoff (> 6.9 U/mL). This OR rule was chosen to maximize sensitivity for detecting colorectal neoplasia, which is a priority for an initial screening test. All screen-positive cases were referred for confirmatory diagnostic colonoscopy.

### Statistical analysis

The primary combined detection strategy was based on the parallel testing (OR rule) described above. Additionally, the diagnostic performance of individual and combined markers (OR rule) was evaluated. Data analysis was performed using SPSS 27.0 software. Continuous data are presented as mean ± standard deviation. Categorical data are presented as frequency (percentage), and differences between groups were analyzed using the chi-square test or Fisher's exact test. The association between positive biomarker tests and CRC was quantified using Odds Ratios (OR) with 95% Confidence Intervals (95% CIs). Diagnostic performance was evaluated by calculating sensitivity, specificity, and their corresponding 95% CIs. To control the risk of Type I errors due to multiple comparisons in subgroup analyses, p-values were adjusted using the Benjamini-Hochberg false discovery rate procedure, with an FDR threshold of 0.05. The significance level was established at 0.05.

## Results

### Baseline characteristics

The baseline characteristics of the 196 participants are shown in Table 1. Overall, there were 100 males (51.0%) and 96 females (49.0%), with a mean age of 58.8 ± 12.0 years. There were no statistically significant differences in age and gender distributions among the groups.

**Table 1:** Baseline characteristics of the study population.

Characteristics	CRC group (n = 65)	AA group (n = 38)	N-AA group (n = 33)	Control group (n = 60)	Overall (n = 196)
Gender (%)					
Male	41 (63.1)	25 (65.8)	12 (36.4)	22 (36.7)	100 (51.0)
Female	24 (36.9)	13 (34.2)	21 (63.6)	38 (63.3)	96 (49.0)
Age (%)					
≤ 39	1 (1.5)	1 (2.6)	1 (3.0)	8 (13.3)	10 (5.1)
40‒49	7 (10.8)	9 (23.7)	4 (12.1)	19 (31.7)	40 (20.4)
50‒59	8 (12.3)	9 (23.7)	6 (18.2)	12 (20.0)	35 (17.9)
60‒69	29 (44.6)	15 (39.5)	15 (45.5)	16 (26.7)	75 (38.3)
≥ 70	20 (30.8)	4 (10.5)	7 (21.2)	5 (8.3)	36 (18.4)
$\bar{x}$±s	63.5 ± 10.5	57.7 ± 10.5	61.9 ± 10.9	52.8 ± 12.5	58.8 ± 12.0

N-AA, Non-Advanced Adenomas; AA, Advanced Adenomas; CRC, Colorectal Cancer. Categorical data were presented as numbers (percentage).

### Diagnostic performance of fecal SDC2 methylation

The diagnostic performance of individual and combined markers is detailed in Table 2. Fecal mSDC2 showed a sensitivity of 86.2% (56/65) and a specificity of 96.7% (58/60) for CRC. It demonstrated high sensitivity for both proximal (91.7%, 22/24) and distal (84.2%, 32/38) colon cancer, and across all stages (I‒IV). As shown in Table 3, the OR for CRC associated with a positive mSDC2 test was 28.9 (95% CI 8.6–97.2, p < 0.001). The sensitivity of the fecal mSDC2 test for CRC was significantly higher than that of CEA or CA72–4, with a statistically significant difference (p < 0.001). Additionally, the detection positivity rate in advanced adenomas was 34.2% (13/38). The detection positivity rate for non-advanced adenomas was 33.3% (11/33). These findings suggest that the test has certain screening value for precancerous lesions, albeit with limited sensitivity for early-stage lesions. Given the limited number of advanced adenomas in this study, these preliminary findings suggest that the test may have certain screening value for precancerous lesions.

**Table 2:** Performance comparison of fecal mSDC2 and blood markers.

Feature	mSDC2	CEA	CA72–4	CEA/CA72–4	mSDC2/CEA/CA72–4
CRC group (%)	86.2 (56/65)	56.9 (37/65)	26.1 (17/65)	69.2 (45/65)	96.9 (63/65)
Tumor site					
Near-end	91.7 (22/24)	70.8 (17/24)	29.2 (7/24)	79.2 (19/24)	100.0 (24/24)
Far-end	84.2 (32/38)	44.7 (17/38)	21.1 (8/38)	60.5 (23/38)	94.7 (36/38)
Stage					
Stages I	75.0 (9/12)	0	33.3 (4/12)	33.3 (4/12)	100.0 (12/12)
Stages II	76.5 (13/17)	47.1 (8/17)	23.5 (4/17)	70.6 (12/17)	94.1 (16/17)
Stages III	91.7 (11/12)	66.7 (8/12)	8.3 (1/12)	66.7 (8/12)	91.7 (11/12)
Stages IV	95.8 (23/24)	87.5 (21/24)	33.3 (8/24)	87.5 (21/24)	100.0 (24/24)
AA group (%)	34.2 (13/38)	10.5 (4/38)	15.8 (6/38)	23.6 (9/38)	50.0 (19/38)
Severe dysplasia	62.5 (5/8)	25.0 (2/8)	50.0 (4/8)	62.5 (5/8)	87.5 (7/8)
≥ 10 mm	28.6 (4 /14)	7.1 (1/14)	7.1 (1/14)	14.3 (2/14)	42.9 (6/14)
Cylindroma/tubular	25.0 (4/16)	6.3 (1/16)	6.3 (1/16)	12.5 (2/16)	37.6 (6/16)
N-AA group (Sensitivity)	33.3 (11/33)	6.0 (2/33)	12.1 (4/33)	15.1 (5/33)	42.3 (14/33)
Control group (Specificity)	96.7 (58/60)	96.7 (58/60)	83.3 (50/60)	81.7 (49/60)	78.3 (47/60)

The combined tests (CEA/CA72–4 and mSDC2/CEA/CA72–4) were based on a parallel testing (OR rule): a test was considered positive if any of the included markers was positive.

mSDC2, SDC2 Methylation; CRC, Colorectal Cancer; AA, Advanced Cdenomas; N-AA, Non-Advanced Adenomas.

**Table 3:** Odds ratios of CRC for fecal mSDC2 and blood index detection.

Classify	mSDC2		CEA		CA72–4	
	OR (95% CI)	p	OR (95% CI)	p	OR (95% CI)	p
CRC (Overall)	28.9 (8.6–97.2)	0.001	5.7 (2.0–16.4)	0.001	2.2 (1.7–2.9)	0.001
Tumor Location						
Proximal	27.5 (7.0–108.0)	0.001	8.3 (4.1–17.0)	0.001	3.4 (2.0–5.9)	0.001
Remote	10.0 (4.7–21.6)	0.001	3.4 (2.3–5.0)	0.001	2.4 (1.5–3.6)	0.001
Cancer Stage						
Stage I/II	8.5 (4.2–17.3)	0.001	3.0 (1.9–4.9)	0.001	3.6 (2.2–5.7)	0.001

mSDC2, SDC2 Methylation; CRC, Colorectal Cancer; OR, Odds Ratio; CI, Confidence Interval.

### Diagnostic performance of serum markers CEA and CA72–4

The sensitivity of serum CEA detection in the CRC group was 56.9% (37/65), and the specificity was 96.7% (58/60), with an OR for serum CEA of 5.7 (95% CI 2.0–16.4, p < 0.001). The CEA levels in the CRC group were significantly higher than those in other groups, with statistical significance (p < 0.001), and a linear correlation was observed (p < 0.001). There were no significant differences in CEA levels among the other groups. The sensitivity of CA72–4 detection for CRC was 26.1% (17/65), and the specificity was 83.3% (50/60), with an OR for CA72–4 of 2.2 (95% CI 1.2–2.9, p < 0.001). The combined detection of CEA/CA72–4 had a sensitivity of 69.2% (45/65) and a specificity of 81.6% (49/60) for CRC. Compared with single detection, the combined detection increased the diagnostic sensitivity for CRC but decreased the specificity (Tables 2 and 3).

### Diagnostic performance of the triple combination

As shown in Tables 2 and 3, the triple combination (mSDC2 + CEA + CA72–4) achieved a sensitivity of 96.9% (63/65) and a specificity of 78.3% (47/60) for CRC. For the N-AA group, the combined test showed a sensitivity of 42.4% (14/33), while it demonstrated a specificity of 78.3% (47/60) in the control group. In subgroup analyses, the combined detection showed numerically higher sensitivity than single detection, with a preliminary observation of 100.0% (24/24) in proximal and 94.7% (36/38) in distal CRC. However, these subgroup analyses, particularly for proximal CRC (n = 24), are underpowered due to limited sample size.

## Discussion

Optimizing the early screening strategies for CRC is an important issue in cancer prevention and control in China.^18^ The progression of CRC is relatively slow, typically undergoing a process from colorectal mucosal lesions to adenomatous polyps and then to malignant tumors.^19^ This process may take several years or even longer, thus providing a golden window period for early detection and diagnosis. Primary prevention through lifestyle modification, secondary prevention through screening for early CRC and significant precancerous lesions, and tertiary prevention through standardized treatment can effectively reduce the incidence and mortality of CRC.^20^^,^^21^

This study confirmed through cohort analysis that fecal SDC2 methylation detection has a sensitivity of 86.2% for CRC, which is comparable to the reported sensitivity of the widely used FIT, which typically ranges from 70% to 80% for CRC but drops significantly for advanced adenomas (approximately 20%–40%).^22^ Moreover, the combination of fecal mSDC2 detection with serum CEA/CA72–4 significantly improved the detection performance, achieving a sensitivity that falls within the previously reported range (91%–94%) for the more advanced FIT-DNA test.^23^^,^^24^

The combined testing strategy in this study employed a parallel approach (OR rule), where a positive result from any of the three markers defined a positive screen. This algorithm was deliberately chosen to maximize sensitivity for CRC detection, a critical attribute for a screening test where missing a true case has severe consequences. While this approach maximized sensitivity for CRC (96.9%), it incurred a practical limitation: a substantial reduction in specificity (from 96.7% with mSDC2 alone to 78.3%). This sensitivity-specificity trade-off is a recognized challenge in developing highly sensitive screening tests, as it directly inflates the false-positive rate and the burden of subsequent diagnostic procedures.^22^^,^^25^ To quantify the impact within a real-world screening context, the Positive Predictive Value (PPV) was calculated. Using the formula PPV = (sensitivity × prevalence) / (sensitivity × prevalence) + (1-specificity) × (1-prevalence), and assuming a CRC prevalence of 0.8%,^11^ the PPV of the combined diagnostic assay was calculated to be approximately 3.5%. This indicates that in such a general screening population, over 96% of individuals with a positive test result would undergo an unnecessary colonoscopy. Even in a higher-risk setting with a prevalence of 5%, the PPV would only reach about 18.2%. Such a high false-positive rate translates into significant healthcare resource utilization, patient anxiety, and procedural risks, which are critical determinants of a screening program's feasibility and cost-effectiveness.^2^ Future research should not only validate these findings in prospective screening cohorts but also explore risk-adapted application to optimize the balance between detection and resource use.

Stool DNA methylation testing is gradually emerging as a novel technology in CRC screening systems due to its non-invasive nature and the convenience of home sampling.^26^ In this study, the sensitivity of mSDC2 testing for CRC was 86.2%, and the detection rate for advanced adenomas was low at 34.2%, which is basically consistent with previous studies. However, this also suggests that relying solely on methylation biomarkers may miss early lesions. In an exploratory, post-hoc analysis, the authors observed that a number of AA and N-AA cases (9/38 and 8/33, respectively) yielded Ct values in a gray zone (38‒39), close to the prespecified positive cutoff. While this observation suggests a potential continuum of SDC2 methylation levels during adenoma progression, it requires validation in future studies with pre-specified criteria for such indeterminate ranges. This phenomenon may be related to the dynamic changes in epigenetic modifications during tumor progression. SDC2 promoter methylation shows a progressive accumulation feature in the adenoma-carcinoma sequence.^27^

This study explored the combined strategy of stool DNA testing and serum markers. Despite the limited sample size in subgroups, the present data suggest that the two types of biomarkers are complementary in terms of biological origin and temporal expression, which can significantly improve the sensitivity to CRC. In terms of biological origin, stool mSDC2 directly reflects the epigenetic abnormalities of colorectal epithelial cells and has specific recognition ability for early carcinogenesis.^28^ In contrast, serum CEA and CA72–4 originate from tumor cell secretion, and their elevated levels are often associated with tumor invasion and metastasis.^10^ Additionally, in the temporal dimension, mSDC2, as an early molecular event in precancerous lesions, can be detected at the adenoma stage.^29^ The dynamic changes of serum markers are positively correlated with tumor progression. This study showed that the sensitivity of CEA for mid-to-late-stage CRC was significantly higher than that for stage I/II (80.0% vs. 27.3%). CA72–4, targeting a different tumor-associated glycoprotein, may capture a subset of CRC cases negative for CEA or mSDC2.^10^ Although its sensitivity for all-stage CRC was low (26.1%), it showed notable sensitivity in Stage I/II cancers (33.3%) and in advanced adenomas with high-grade dysplasia (50%). In the CEA/CA72–4 panel, CA72–4 contributed to the detection of 8 additional CRC cases beyond CEA alone, raising the combined sensitivity from 56.9% to 69.2%. This marginal gain, however, came at the cost of reduced specificity, illustrating the trade-off inherent in panel expansion. The inclusion of CA72–4 in the triple-panel was therefore exploratory, based on its potential to detect biologically distinct subsets and provide complementary value within a multi-analyte strategy.^10^^,^^30^ Its definitive utility requires further validation, weighing the marginal sensitivity gain against the confirmed specificity cost.

The present study has the following limitations: First, the case-control design with hospital-based recruitment is susceptible to spectrum bias. The control group, comprising individuals who underwent colonoscopy, likely represents a subset with a higher pre-test probability of disease or specific symptoms/family history compared to a true asymptomatic screening population. Therefore, the performance of this test, particularly its specificity, requires further validation in a prospective, population-based screening cohort to confirm its real-world applicability. Second, fecal immunochemical testing was not included for parallel comparison, which limited the assessment of effectiveness compared with traditional screening methods. Future studies designed as head-to-head comparisons within a large, prospective screening cohort are essential to validate its true clinical utility relative to FIT and FIT-DNA. Third, the overall sample size, particularly for subgroup analyses, is limited. This results in underpowered analyses and wide confidence intervals for sensitivity estimates. Therefore, these subgroup findings should be viewed as exploratory and preliminary, requiring validation in larger, adequately powered studies. Future studies could verify the generalizability through multicenter screening cohorts and explore the integration and optimization of multimodal data using machine learning algorithms.

## Conclusion

In summary, the non-invasive combination of fecal mSDC2 with serum CEA and CA72–4 showed potential to effectively increase the detection rate of CRC. The combined approach has the advantages of being non-invasive and convenient. While the findings for advanced adenomas are promising, they are based on a small sample and require further verification. Future large-scale prospective studies are needed to confirm the clinical utility and generalizability of this combined strategy before broad clinical promotion can be recommended.

## Ethics approval and consent to participate

The study protocol was approved by the Institutional Ethics Board of the Civil Aviation General Hospital, Beijing, China n°2023-l-K-07). Due to the retrospective design of the study and the fact that the study evaluated clinical practice, the Institutional Ethics Board of the Civil Aviation General Hospital waived the need for individual informed consent.

## Data availability statement

The datasets used and/or analyzed during the current study are available from the corresponding author on reasonable request.

## Authors' contributions

Zhen Li: Conceptualization; methodology; formal analysis; investigation; data curation; writing-original draft; writing review & editing.

Jianping Cheng: Methodology; software; formal analysis; investigation; writing-original draft; writing-review & editing.

Xiaolin Zhao: Investigation; data curation; writing-review & editing.

Yang Liu: Conceptualization; data curation; writing-review & editing.

Yanqiong Zhang: Formal analysis; investigation; writing-review & editing.

Yanping Li: Formal analysis; investigation; writing-review & editing.

Yixiao Zhao: Investigation;writing-review & editing.

Chanjuan Fan: Conceptualization; formal analysis; writing-review & editing.

## Funding

This research did not receive any specific grant from funding agencies in the public, commercial, or not-for-profit sectors.

## Declaration of competing interest

The authors declare no conflicts of interest.

## References

[1] Jacobsson M., Wagner V., Kanneganti S. (2024). Screening for colorectal cancer. Surg Clin North Am.

[2] Chung D.C., Gray D.M., Singh H., Issaka R.B., Raymond V.M., Eagle C. (2024). A cell-free DNA blood-based test for colorectal cancer screening. N Engl J Med.

[3] Underwood P.W., Ruff S.M., Pawlik T.M. (2024). Update on targeted therapy and immunotherapy for metastatic colorectal Cancer. Cells.

[4] Allemani C., Matsuda T., Di Carlo V., Harewood R., Matz M., Nikšić M., et al.; CONCORD Working Group. Global surveillance of trends in cancer survival 2000-14 (CONCORD-3): analysis of individual records for 37513025 patients diagnosed with one of 18 cancers from 322 population-based registries in 71 countries. Lancet. 2018;391(10125):1023–75. 10.1016/S0140-6736(17)33326-3 PMC5879496 29395269

[5] Siegel R.L., Wagle N.S., Cercek A., Smith R.A., Jemal A. (2023). Colorectal cancer statistics, 2023. CA Cancer J Clin.

[6] Davidson K.W., Barry M.J., Mangione C.M., Cabana M., Caughey A.B., Davis E.M. (2021). Screening for colorectal cancer: us preventive services task force recommendation statement. JAMA.

[7] Expert Group on Early Diagnosis and Treatment of Cancer (2023). Chinese society of oncology, Chinese medical association. [Expert consensus on the early diagnosis and treatment of colorectal cancer in China (2023 edition)]. Zhonghua Yi Xue Za Zhi.

[8] Zhao S., Wang S., Pan P., Xia T., Wang R., Cai Q. (2022). Gastrointestinal early cancer prevention & treatment alliance of China (GECA). FIT-based risk-stratification model effectively screens colorectal neoplasia and early-onset colorectal cancer in Chinese population: a nationwide multicenter prospective study. J Hematol Oncol.

[9] Iacuzzo C., Germani P., Troian M., Mis T.C., Giudici F., Osenda E. (2021). Serum carcinoembryonic antigen pre-operative level in colorectal cancer: revisiting risk stratification. ANZ J Surg.

[10] Kildusiene I., Dulskas A., Smailyte G. (2024). Value of combined serum CEA, CA72-4, and CA19-9 marker detection in diagnosis of colorectal cancer. Tech Coloproctol.

[11] Chen H., Li N., Ren J., Feng X., Lyu Z., Wei L. (2019). Group of cancer screening program in urban China (CanSPUC). Participation and yield of a population-based colorectal cancer screening programme in China. Gut.

[12] Raut J.R., Guan Z., Schrotz-King P., Brenner H. (2020). Fecal DNA methylation markers for detecting stages of colorectal cancer and its precursors: a systematic review. Clin Epigenetics.

[13] Li N., Li C., Zhang X., Wang F., Li L., Liang J. (2023). Diagnostic value of human fecal SDC2 gene in colorectal cancer. Am J Transl Res.

[14] Wang J., Liu S., Wang H., Zheng L., Zhou C., Li G. (2020). Robust performance of a novel stool DNA test of methylated SDC2 for colorectal cancer detection: a multicenter clinical study. Clin Epigenetics.

[15] Zhao S., He Z., Sui X., Zhang S., Li Z., Bai Y. (2024). Real-world stool-based Syndecan-2 Methylation test improved detection of advanced colorectal neoplasia for colorectal cancer screening: a prospective, multicenter, community-based study. Gastroenterology.

[16] Young G.P., Benton S.C., Bresalier R.S., Chiu H.-M., Dekker E., Fraser C.G. (2025). Fecal immunochemical test positivity thresholds: an international survey of population-based screening programs. Dig Dis Sci.

[17] Ma L., Qin G., Gai F., Jiang Y., Huang Z., Yang H. (2022). A novel method for early detection of colorectal cancer based on detection of methylation of two fragments of syndecan-2 (SDC2) in stool DNA. BMC Gastroenterol.

[18] Yang Y., Gao Z., Huang A., Shi J., Sun Z., Hong H. (2023). Epidemiology and early screening strategies for colorectal cancer in China. Chin J Cancer Res.

[19] Cubiella J., Regueiro-Expósito C. (2023). Colorectal cancer: from prevention to treatment. Best Pract Res Clin Gastroenterol.

[20] Betesh A.L., Schnoll-Sussman F.H. (2021). Colorectal cancer screening in the elderly. Clin Geriatr Med.

[21] Peterse E.F.P., Meester R.G.S., Siegel R.L., Chen J.C., Dwyer A., Ahnen D.J. (2018). The impact of the rising colorectal cancer incidence in young adults on the optimal age to start screening: microsimulation analysis I to inform the American cancer society colorectal cancer screening guideline. Cancer..

[22] Meklin J., Syrjänen K., Eskelinen M. (2020). Colorectal cancer screening with traditional and new-generation fecal immunochemical tests: a critical review of fecal occult blood tests. Anticancer Res.

[23] Li J., Yao H., Lu Y., Zhang S., Zhang Z. (2024). Chinese national clinical practice guidelines on prevention, diagnosis and treatment of early colorectal cancer. Chin Med J (Engl).

[24] Dolatkhah R., Dastgiri S., Jafarabadi M.A., Abdolahi H.M., Somi M.H. (2022). Diagnostic accuracy of multitarget stool DNA testing for colorectal cancer screening: a systematic review and meta-analysis. Gastroenterol Hepatol.

[25] Berger B.M., Levin B., Hilsden R.J. (2016). Multitarget stool DNA for colorectal cancer screening: a review and commentary on the United States preventive services draft guidelines. World J Gastrointest Oncol.

[26] Porcaro F., Voccola S., Cardinale G., Porcaro P., Vito P. (2024). DNA methylation biomarkers in stool samples: enhancing colorectal cancer screening strategies. Oncol Rev.

[27] Siri G., Alesaeidi S., Dizghandi S.E., Alani B., Mosallaei M., Soosanabadi M. (2022). Analysis of SDC2 gene promoter methylation in whole blood for noninvasive early detection of colorectal cancer. J Cancer Res Ther.

[28] Song J.H., Oh T.J., An S., Lee K.H., Kim J.Y., Kim J.S (2023). Comparative detection of syndecan-2 methylation in preoperative and postoperative stool DNA in patients with colorectal cancer. World J Gastrointest Surg.

[29] Zhang L., Wang D., Li Z., Lin G., Li J., Zhang R. (2024). Interlaboratory consistency of SDC2 promoter methylation detection in colorectal cancer using the post-optimized materials. iScience.

[30] Ladabaum U., Mannalithara A., Meester R.G.S., Gupta S., Schoen R.E. (2019). Cost-effectiveness and national effects of initiating colorectal cancer screening for average-risk persons at age 45 years instead of 50 Years. Gastroenterology.

